# Morbidity patterns and survival among children living with HIV and HIV-exposed uninfected children in North Karnataka, India: a community-based prospective cohort study

**DOI:** 10.3389/fpubh.2026.1823720

**Published:** 2026-10-02

**Authors:** Ashok S. Dorle, Mubashir Angolkar, R. Manjula, Jayaraj Mhetri, Mayank Singh, Nagaraj B. Patil, Sukanya Sarma, Prabhu Bilagi, Rajeev Sethumadhavan, Anju Sinha

**Affiliations:** 1 Department of Community Medicine, S. Nijalingappa Medical College, Bagalkot, Karnataka, India; 2 School of Public Health, KLE Academy of Higher Education and Research (KAHER), Belagavi, Karnataka, India; 3 Deparment of Biostatistics and Health Data Science, KLE Academy of Higher Education and Research (KAHER), Belagavi, Karnataka, India; 4 Indian Council of Medical Research (ICMR), New Delhi, India; 5 Magic Bus Foundation, Mumbai, India; 6 National TB Elimination Program, WHO Country Office for India, New Delhi, India

**Keywords:** antiretroviral therapy, children living with HIV, cohort study, morbidity, paediatric HIV

## Abstract

**Background:**

Children living with HIV (CLHIV) continue to face high rates of infection and morbidity despite improved ART coverage. This study aimed to (i) compare the prevalence and factors associated with acute morbidities between CLHIV and HIV-exposed uninfected children (HEUC) and (ii) estimate survival probabilities over a two-year follow-up period in a community-based cohort from North Karnataka.

**Materials and methods:**

This prospective cohort study was conducted in Belagavi district. Data were collected over eight visits between April 2022 and March 2024. Ethical approval was obtained from the Institutional Ethics Committee of KAHER and SNMC, and permission was granted by the Karnataka State AIDS Prevention Society (KSAPS). Multivariable logistic regression was used to assess the association between HIV status and acute morbidities at baseline and endline, adjusting for sociodemographic factors. Survival probabilities were estimated using the Kaplan–Meier method and log-rank test.

**Results:**

A total of 348 children (184 CLHIV and 164 HEUC) with complete baseline and endline data were included in the morbidity analysis, whereas 372 children who were followed for their survival were included in the survival analysis. CLHIV were generally older and more often male. Among CLHIV, 19.0% had lost both parents, compared to 6.1% among HEUC. CLHIV showed higher gastrointestinal and skin morbidity. The adjusted odds of morbidity were ~2-fold at baseline and ~3-fold at the endline. Survival remained high (99% at 1 year and 98% at 2 years), with four deaths recorded.

**Conclusion:**

This study demonstrates that CLHIV in North Karnataka experience a substantial burden of morbidity. These health challenges are compounded by social factors, such as orphanhood and limited school attendance. The longitudinal design provides important findings to guide policy decisions and health interventions for vulnerable populations.

## Introduction

The incidence and prevalence of paediatric HIV in India present a significant public health challenge, with a considerable number of children affected by the virus (1). Early detection of HIV in children is crucial for mitigating morbidity, growth delays, and mortality; however, India has historically lacked comprehensive epidemiological data on this disease burden (1, 2). The National AIDS Control Program (NACP) estimates paediatric HIV cases as a proportion of adult cases; however, the high mortality rates among Children living with HIV (CLHIV), with one-third dying before their first birthday and half before their second, highlight the urgent need for more accurate and timely data (1). For instance, a community-based study conducted in Belagavi district, Karnataka, between 2014 and 2018, aimed to address this gap by estimating the HIV incidence and prevalence, as well as associated risk factors among children exposed to maternal HIV (3). This region is particularly relevant given Karnataka’s higher HIV prevalence compared to the national average, with children constituting approximately 6.3% of all people living with HIV (PLHIV) in the state (4).

Since the first description of human immunodeficiency virus (HIV) infection in Central Africa, the epidemic has become a major global public health challenge (5, 6). According to 2023 data, India accounts for 17.8% of the world’s population and 5.5% of the global PLHIV burden (7–9). Children aged less than 15 years accounted for approximately 1.7 million (4.5%) of all PLHIV (7, 9). Among PLHIV in India, 2.85% were children. An estimated 63,000 new HIV infections occur in India each year, where children aged 0–14 years account for 8%. The epidemic in India is concentrated in key populations, which, through mother-to-child transmission and parental morbidity/mortality, indirectly impacts the vulnerability of children to HIV infection and its consequences (10). Karnataka is a high-prevalence state in South India, accounting for 4.6% of the national population and approximately 11.5% of India’s PLHIV. CLHIV accounts for approximately 6.8% of PLHIV in the state. Between 2005 and 2017, India had a drop in new HIV infections; however, the decline in paediatric infections was more pronounced than that of adults, which was largely attributable to the Prevention of Parent-to-Child Transmission (PPTCT) program. Nonetheless, gaps in antenatal registration, HIV testing coverage, antiretroviral (ARV) prophylaxis, and linkage to care remain (10–12). Despite improvements, several structural and social barriers, including incomplete coverage of antenatal HIV testing, underreporting of private-sector HIV services, nonadherence to ART, stigma, and poor health system linkages, continue to increase the risk of infection and poor outcomes among children born to mothers living with HIV (7, 10, 11, 13, 14). Infectious diseases remain the most frequently reported comorbidities among CLHIV. Tuberculosis (TB) is a leading cause of morbidity and mortality in HIV-infected children, particularly in settings with high TB prevalence (15).

These persistent gaps and barriers, particularly in a high-prevalence state like Karnataka, underscore an unmet need for contemporary, robust data on how these systemic and social challenges influence the day-to-day morbidity burden and long-term survival of CLHIV. There is a lack of recent, comprehensive longitudinal data from this specific region to evaluate the effectiveness of current interventions and identify residual vulnerabilities that impact these children’s health over time. To address these gaps, the Indian Council of Medical Research (ICMR) implemented a Task Force project on paediatric HIV in the Belagavi district of Karnataka in two phases (2011–2014 and 2014–2018). They identified a cohort of 2,022 HIV-exposed children (230 HIV-infected, 1,694 HIV-uninfected, and 98 untested) from 977 families across the district. Such studies are vital for informing public health strategies, especially given the rising trend of HIV infection in pregnant women. While antiretroviral therapy (ART) is freely available for children at government centers in India, assessing the survival outcomes of children with good ART adherence remains important (1, 2). Although these earlier studies (Phases I and II) successfully estimated the burden of paediatric HIV and MTCT risks (2, 3), they did not comprehensively assess the longitudinal forms and their evolving determinants or long-term survival outcomes within the specific cohort following the implementation of widespread ART and PPTCT. Additionally, the persistent structural and social barriers identified (e.g., inadequate antenatal HIV testing, nonadherence to ART, stigma, and poor health system linkages) suggest an ongoing need to understand their effect on the concrete health trajectories of CLHIV and HIV-exposed uninfected children (HEUC) in this region.

The present study represents Phase III of this longitudinal effort (2022–2024), which was designed to revive and follow the established paediatric cohort. Explicitly, detailed insights into the shifting spectrum of morbidities, the enduring or emerging determinants of these morbidities, and the actual survival rates under current program conditions are largely unquantified. Such data are crucial for identifying persistent health disparities and tailoring effective, evidence-based paediatric HIV programming and policies, an unmet need for children in similar resource-limited settings.

## Data and methods

### Study design and setting

We conducted a prospective community-based cohort study in the Belagavi district of Karnataka, India. It shares borders with Maharashtra (Sangli and Kolhapur districts), Goa, and multiple districts in Karnataka. Administratively, the district comprises 10 administrative sub-districts (talukas), which were grouped into two operational sites for this study. The study site for each taluka is discussed in Table 1.

**Table 1:** Distribution of study sites across talukas in Belagavi District, Karnataka, India.

Sites by talukas for the study	
Site 1	Site 2
Ramdurg	Belagavi
Chikodi	Hukkeri
Gokak	Khanapur
Athani	Bailhongal
Raibag	Saudatti

### Cohort background and study population

The cohort for this phase III study was derived from the earlier ICMR Task Force project “Estimating the burden of paediatric HIV in an ‘A’ category district in India” (1). Phase I of that project used three complementary strategies: pregnant women, HIV-positive parents, and sick children attending health facilities to estimate the burden of paediatric HIV among children aged 0–15 years in the Belagavi district (1, 2). Phase II followed the identified children longitudinally to estimate HIV incidence, describe patterns and factors associated with morbidities, assess nutritional status, and measure mortality. By the end of Phase II, the study population included 291 health facilities, 985 households, and 2,022 children (230 HIV-infected, 1,694 HIV-uninfected, and 98 untested children). All participants were originally enrolled during childhood and were prospectively followed up through successive study phases. At the time of the current assessment (April 2022–March 2024), some cohort members had transitioned into adulthood (aged ≥18 years). They were retained in the study as part of the original paediatric cohort to ensure continuity of longitudinal follow-up.

This Phase III study (2022–2024) aimed to:

- Trace and re-establish the existing paediatric cohort identified during Phases I and II in Belagavi district.
- To prospectively follow the cohort at 3-month intervals over 24 months (eight follow-up visits) to document morbidity and mortality outcomes, and to compare the morbidity profile between baseline (Visit 1) and the final follow-up (Visit 8).

### Eligibility criteria

Children were eligible for inclusion if they had been enrolled in Phases I and/or II of the ICMR paediatric HIV study in the Belagavi district, were residing in any taluka of the district at the time of Phase III, and had parents or guardians who were willing to provide written informed consent for participation, including consent for age-appropriate blood testing.

### Sample size and sampling

#### Sample size was calculated using the formula

The sample size was calculated using the standard formula for estimating a proportion. The *z* value was set at 1.96 for a 95% confidence interval, and proportion (*p*) was 63%. This estimate was based on reported antiretroviral therapy (ART) coverage among children living with HIV (CLHIV), as reported by UNAIDS and NACO. An absolute precision (*d*) of 7% was considered. Based on these assumptions, the required sample size for CLHIV was estimated to be 183 participants. Assuming a 1:1 ratio between CLHIV and HIV-exposed uninfected children (HEUC), a corresponding sample of 183 participants was required in the HEUC group.

Considering these requirements and the availability of participants in the established cohort, we targeted all 230 CLHIV and an equal number of HEUC from the existing cohort for this study. All CLHIV and their HIV-uninfected siblings enrolled during Phases I and II were contacted. Subsequently, all eligible participants from the established cohort were approached for inclusion in Phase III. The final number of participants included in the morbidity and survival analyses is presented in the Results section and illustrated in Figure 1.

**Figure 1:**
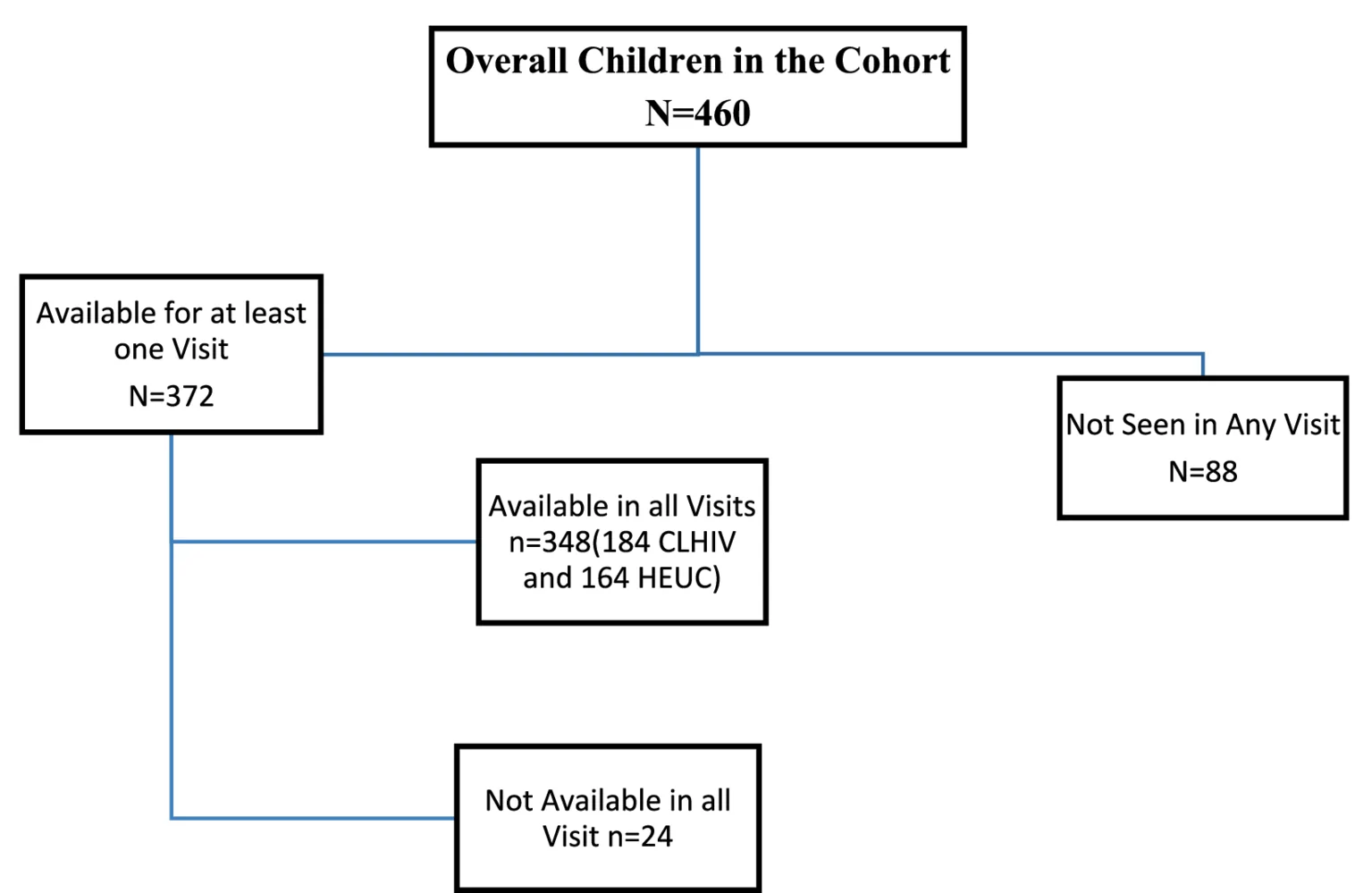
Flow diagram illustrating the recruitment and selection of study participants included in the prospective paediatric HIV cohort analysis. CLHIV-Children living with HIV, HEUC-HIV Exposed Uninfected Children.

### Data collection procedure

Four field investigators (FIs) were trained in standardized data collection procedures through initial and refresher training. Using a line list generated from previous phases, FIs contacted families at their residences or other convenient and confidential locations. After obtaining written informed consent from parents/guardians (and assent where appropriate), the children were enrolled in Phase III during the first visit, which was considered the baseline. Each child was followed up at three-month intervals for up to eight visits from April 2022 to March 2024. At each follow-up visit, data were collected using pretested tools and included sociodemographic characteristics, clinical symptoms, and signs with a focus on acute morbidities affecting the respiratory, gastrointestinal, skin, and other systems and HIV-related information, including antiretroviral therapy status and adherence where applicable. Morbidity comparisons were performed between baseline (Visit 1) and final follow-up (Visit 8) to evaluate overall changes during the study period. Mortality was monitored throughout the follow-up visits.

Clinical examinations and anthropometric measurements were performed at each visit using standard procedures. For HEUC, HIV status was confirmed using age-appropriate rapid antibody tests at baseline and endline, following national guidelines, to ensure their continued uninfected status.

TB-related information was assessed among children enrolled in the prospective paediatric HIV cohort. For children diagnosed with TB during follow-up, information on the number of TB episodes, type of TB (pulmonary or extrapulmonary), presenting symptoms, diagnostic investigations, and microbiological confirmation was obtained from clinical records and follow-up assessments.

### Outcomes and variables

#### Primary outcomes

The primary outcomes were the presence of specific categories of acute morbidity during the defined recall period (the last three months), including other conditions such as ear discharge and oral lesions (e.g., candidiasis), and non-specific complaints such as generalized weakness, persistent fever of unknown origin, or neurological symptoms. Acute morbidity was classified into five categories (fever, gastrointestinal symptoms, respiratory symptoms, skin conditions, and others). The others category included acute symptoms such as eye discharge, ear discharge, mouth ulcer, and oral candidiasis. A composite outcome, termed “any morbidity,” was defined as the presence of one or more acute morbidities in any of the five categories mentioned above. Survival outcomes were assessed by documenting survival status and time to death over the follow-up period.

#### Secondary outcomes

Secondary outcomes included the prevalence of specific categories of acute morbidity, such as fever, diarrhea, respiratory symptoms, and skin conditions, assessed at baseline and endline, as well as sociodemographic factors associated with these morbidities. Nutritional indicators and anthropometric measurements were also collected and, where applicable, considered in the analysis; however, these findings are not presented in detail in this report.

#### Key explanatory variables

Key explanatory variables included the child’s HIV status (CLHIV or HEUC), age group (5–9, 10–14, 15–19, and ≥20 years), and sex. Household and caregiving characteristics included parental survival status (both parents alive, mother deceased, father deceased, or both deceased), primary caregiver/support person (mother, father, guardian, or others/warden), living arrangement (with mother at home, without mother at home, or in a hostel, other home, or orphanage), family type (nuclear or joint), and the Number of household/family members living with participant (≤4 or >4). Social and economic variables included school or college attendance (yes or no), marital status (married or unmarried), and household wealth, which was categorized into wealth index quintiles (Q1–Q5), where Q1 represented the poorest quintile, and Q5 represented the wealthiest quintile of the study population.

### Statistical analysis

Data were coded in Excel and analyzed using SPSS v19. Quantitative variables are presented as mean ± SD and qualitative variables as frequencies and percentages. We used multivariable logistic regression to estimate adjusted odds ratios (AORs) for HIV status and relevant sociodemographic covariates. Covariates included in the multivariable logistic regression models were selected *a priori* based on established evidence from the literature and their potential role as confounders, rather than by statistical significance or automated variable selection procedures. A consistent adjustment set was applied across models to facilitate comparability of effect estimates. We set statistical significance at *p* < 0.05. Time-to-event analyses were performed using the Kaplan–Meier method, with time origin defined as the date of enrolment into Phase III (Visit 1). Time was measured in months from enrolment until death or censoring. Participants who remained alive at the end of follow-up or were lost to follow-up were treated as censored observations at their last known follow-up. Survival distributions between HIV status groups were compared using the log-rank test. The primary comparative analysis compared morbidity status at baseline (Visit 1) with morbidity status at the final follow-up (Visit 8). The analysis was designed to assess the change in morbidity between the beginning and end of the follow-up period; intermediate visits were not included in this comparative analysis.

### Ethical considerations

Ethical approval was obtained from the Institutional Ethics Committees of S. N. Medical College and KAHER Belagavi. Written informed consent was obtained from the parents/guardians, with assent from older children, as per the guidelines. Participants aged 18 years and above provided written informed consent before enrolment in the follow-up assessment. Permission to access the program data and cohort tracing support was provided by the Karnataka State AIDS Prevention Society (KSAPS).

## Results

### Participant characteristics

The taluka-wise distribution of the study participants (*n* = 348) is summarized in Table 2. Gokak contributed the largest share (23.9%), followed by Belagavi (16.4%) and Athani (12.6%).

**Table 2:** Distribution of children by taluka in the prospective paediatric HIV cohort included in the analysis, Belagavi District, Karnataka, India (*N* = 348).

Taluka	*n* (%)
Bailhongal	29 (8.3)
Athani	44 (12.6)
Belagavi	57 (16.4)
Chikkodi	20 (5.7)
Gokak	83 (23.9)
Hukkeri	19 (5.5)
Khanapur	23 (6.6)
Savadatti	31 (8.9)
Raibag	29 (8.4)
Ramdurg	13 (3.7)
Total	348 (100.0)

Baseline characteristics (Table 3) showed notable differences between CLHIV and HEUC groups. CLHIV were predominantly older, with 37.5% in both the 10–14 and 15–19 year groups, while HEUC were mainly 10–14 years (43.3%). Males constituted 59.2% of CLHIV, whereas females comprised 56.7% of the HEUCs. Parental loss was more common among CLHIV, with only 28.3% having both parents alive compared to 46.3% of HEUCs. Consequently, more CLHIV were under guardian care (15.8% vs. 7.3%) and living in hostels (10.9% vs. 1.8%). School attendance was lower among CLHIV (64.1%) than among NCs (79.3%). Wealth distribution was similar across groups, although CLHIV had a slightly higher representation in the richest quintile (17.9% vs. 14.6%). Overall, CLHIV exhibited greater social vulnerability, marked by higher orphanhood and institutional care and lower school attendance.

**Table 3:** Sociodemographic and clinical characteristics of children enrolled in the prospective paediatric HIV cohort at baseline (*N* = 348).

Background characteristics	CLHIV	HEUC
	*n* (%)	*n* (%)
Age		
5–9	23 (12.5)	38 (23.2)
10–14	69 (37.5)	71 (43.3)
15–19	69 (37.5)	45 (27.4)
20+	23 (12.5)	10 (6.1)
Sex		
Male	109 (59.2)	71 (43.3)
Female	75 (40.8)	93 (56.7)
Parental survival status		
Mother and father alive	52 (28.3)	76 (46.3)
Mother deceased	11 (6.0)	19 (11.6)
Father deceased	86 (46.7)	59 (36.0)
Both mother and father deceased	35 (19.0)	10 (6.1)
Primary caregiver/support person		
Mother	129 (70.1)	133 (81.2)
Stepmother	1 (0.5)	1 (0.6)
Father	7 (3.8)	15 (9.1)
Guardian	29 (15.8)	12 (7.3)
Others (warden)	18 (9.8)	3 (1.8)
Living arrangement		
With mother at home	129 (70.1)	132 (80.5)
Without mother at home	28 (15.2)	26 (15.9)
Hostel	20 (10.9)	3 (1.8)
In other home	6 (3.3)	0 (0)
Orphanage	1 (0.5)	3 (1.8)
Type of family		
Nuclear	149 (81.0)	139 (84.8)
Joint	29 (15.7)	23 (14.0)
NA	6 (3.3)	2 (1.2)
Number of household/family members living with participant		
Less or equal 4	117 (63.6)	63 (38.4)
Above 4	67 (36.4)	101 (61.6)
Currently attending school/college		
Yes	118 (64.1)	130 (79.3)
No	66 (35.9)	34 (20.7)
Marital status		
Married	7 (3.8)	9 (5.5)
Unmarried	177 (96.2)	155 (94.5)
Wealth index		
Q1 (Poorest)	55 (30.0)	49 (29.9)
Q2 (Poorer)	17 (9.2)	29 (17.7)
Q3 (Middle)	47 (25.5)	35 (21.3)
Q4 (Richer)	32 (17.4)	27 (16.5)
Q5 (Richest)	33 (17.9)	24 (14.6)
Total	184 (100.0)	164 (100.0)

CLHIV-Children living with HIV, HEUC-HIV Exposed Uninfected Children, NA-Not Applicable.

At Visit 1, fever was reported in 17.4% of CLHIV and 15.2% of HEUC, compared with 15.8 and 11.6%, respectively, at Visit 8. Diarrhoea was less prevalent at Visit 8 than at Visit 1 among CLHIV (8.2% vs. 10.9%), whereas it was more prevalent among HEUC (3.0% vs. 0.6%). Vomiting was less prevalent at Visit 8 in both CLHIV (3.8% vs. 5.4%) and HEUC (1.2% vs. 2.4%). Rhinitis was more prevalent at Visit 8 among both CLHIV (33.7% vs. 29.3%) and HEUC (27.4% vs. 26.8%). Cough was slightly more prevalent among CLHIV at Visit 8 (20.7% vs. 19.6%), while it was less prevalent among HEUC (7.9% vs. 9.1%). Mouth ulcers were more frequently reported among CLHIV at Visit 8 than at Visit 1 (25.0% vs. 17.9%), while no cases were reported among HEUC at Visit 8. Skin rashes and sore throat showed variations between the two assessment points, with skin rashes being slightly less prevalent among CLHIV at Visit 8 (7.6% vs. 9.2%) and sore throat being more prevalent (8.2% vs. 3.3%). Overall, these findings describe differences in morbidity prevalence between Visit 1 and Visit 8 and should not be interpreted as representing continuous trends across the intervening visits (Table 4).

**Table 4:** Prevalence of acute morbidity symptoms among CLHIV and HEUC at baseline (Visit 1) and final follow-up (Visit 8).

Acute morbidity	CLHIV visit 1 *n* (%)	CLHIV visit 8 *n* (%)	Change^*^	HEUC visit 1 *n* (%)	HEUC visit 8 *n* (%)	Change^*^
Fever	32 (17.4)	29 (15.8)	−1.6	25 (15.2)	19 (11.6)	−3.6
Diarrhoea	20 (10.9)	15 (8.2)	−2.7	1 (0.6)	5 (3.0)	+2.4
Vomiting	10 (5.4)	7 (3.8)	−1.6	4 (2.4)	2 (1.2)	−1.2
Worm infestation	0 (0)	0 (0)	0.0	2 (1.2)	0 (0)	−1.2
Rhinitis	54 (29.3)	62 (33.7)	+4.4	44 (26.8)	45 (27.4)	+0.6
Cough	36 (19.6)	38 (20.7)	+1.1	15 (9.1)	13 (7.9)	−1.2
Sore throat	6 (3.3)	15 (8.2)	+4.9	17 (10.4)	13 (7.9)	−2.5
Breathlessness	1 (0.5)	0 (0)	−0.5	0 (0)	0 (0)	0.0
Skin rashes	17 (9.2)	14 (7.6)	−1.6	5 (3.0)	4 (2.4)	−0.6
Tinea capitis	0 (0)	1 (0.5)	+0.5	0 (0)	0 (0)	0.0
Chickenpox	0 (0)	0 (0)	0.0	1 (0.6)	0 (0)	−0.6
Ear discharge	10 (5.4)	8 (4.3)	−1.1	0 (0)	1 (0.6)	+0.6
Mouth ulcers	33 (17.9)	46 (25.0)	+7.1	1 (0.6)	0 (0)	−0.6
Oral candidiasis	1 (0.5)	0 (0)	−0.5	0 (0)	0 (0)	0.0
Others	2 (1.1)	10 (5.4)	+4.3	0 (0)	7 (4.3)	+4.3

CLHIV, Children living with HIV; HEUC, HIV-exposed uninfected children. ^*^Change represents the absolute difference in prevalence between Visit 8 and Visit 1 (V8−V1), expressed in percentage points. Positive values indicate a higher prevalence at Visit 8, while negative values indicate a lower prevalence at Visit 8.

Children living with HIV (CLHIV) had a consistently higher prevalence of acute morbidity than HIV-exposed uninfected children (HEUC) at both the baseline (Visit 1) and endline (Visit 8) assessments (Table 5). Gastrointestinal symptoms were more prevalent in CLHIV at Visit 1 (13.0% vs. 4.3%; UOR 3.36, 95% CI: 1.41–8.03, <0.05) and Visit 8 (10.9% vs. 4.3%; UOR 2.74, 95% CI: 1.13–6.65, <0.05). Skin conditions were also higher among CLHIV at Visit 1 (9.2% vs. 3.7%; UOR 2.68, *p* = <0.05) and Visit 8 (7.6% vs. 2.4%; UOR 3.29, <0.05). The greatest difference was observed in ‘other conditions’, with CLHIV showing markedly higher prevalence at Visit 1 (22.8% vs. 0.6%; UOR 48.21, *p* < 0.001) and Visit 8 (29.4% vs. 4.9%; UOR 8.10, *p* < 0.001). No significant differences were seen for fever or respiratory symptoms (*p* > 0.05). Overall, CLHIV had consistently higher prevalence and odds of gastrointestinal, skin, and other morbidities than HEUC.

**Table 5:** Prevalence and unadjusted odds ratios of acute morbidity conditions according to HIV status at baseline and end-line visits in the prospective paediatric HIV cohort.

Morbidity conditions	Visit 1				Visit 8			
	Ref: absence of the respective morbidity							
	CLHIV	HEUC	UOR	*p* value	CLHIV	HEUC	UOR	*p* value
Fever	32 (17.4)	25 (15.2)	1.17 [0.66, 2.07]	0.54	29 (15.8)	19 (11.6)	1.43 [0.77, 2.66]	0.261
Gastrointestinal symptoms	24 (13.0)	7 (4.3)	3.36^**^ [1.41, 8.03]	<0.05	20 (10.9)	7 (4.3)	2.74^*^ [1.13, 6.65]	<0.05
Respiratory symptoms	67 (36.4)	60 (36.6)	0.99 [0.64, 1.54]	0.973	78 (42.4)	58 (35.4)	1.35 [0.87, 2.07]	0.18
Skin conditions	17 (9.2)	6 (3.7)	2.68^*^ [1.03, 6.97]	<0.05	14 (7.6)	4 (2.4)	3.29^*^ [1.06, 10.22]	<0.05
Others	42 (22.8)	1 (0.61)	48.21^***^ [6.55, 354.77]	<0.05	54 (29.4)	8 (4.9)	8.10^***^ [3.72, 17.64]	<0.05

*p* values are indicated as follows: one asterisk (*) denotes statistical significance at the 95% confidence level (*p* < 0.05), two asterisks (**) denote statistical significance at the 99% confidence level (*p* < 0.01), and three asterisks (***) denote statistical significance at the 99.99% confidence level (*p* < 0.001).

UOR, Unadjusted Odds Ratio; CLHIV, Children living with HIV; HEUC, HIV Exposed Uninfected Children.

After adjustment for sociodemographic covariates, CLHIV had higher adjusted odds of several acute morbidity conditions than HEUC at both assessment points (Table 6). Fever showed no significant difference at Visit 1 (AOR 1.45, *p* = 0.256) and at Visit 8 (AOR 1.84, *p* = 0.084). Gastrointestinal symptoms were consistently higher among CLHIV at Visit 1 (AOR 3.97, <0.05) and Visit 8 (AOR 3.35, <0.05). Respiratory symptoms were significant only at Visit 8 (AOR 1.74, <0.05). Skin conditions became significant at Visit 8 (AOR 3.69, <0.05). The highest odds were observed for ‘other conditions’ at both Visit 1 (AOR 53.81, *p* < 0.001) and Visit 8 (AOR 11.95, *p* < 0.001). Overall, CLHIV had greater adjusted odds of gastrointestinal, respiratory, skin, and other morbidities, particularly at Visit 8, with ‘other conditions’ showing the largest effect.

**Table 6:** Adjusted odds ratios for acute morbidity conditions according to HIV status at baseline and end-line visits in the prospective paediatric HIV cohort.

Morbidity conditions	Visit 1		Visit 8	
	Ref: absence of the respective morbidity			
	AOR	*p* value	AOR	*p* value
Fever	1.45 [0.77, 2.73]	0.256	1.84 [0.92, 3.67]	0.084
Gastrointestinal symptoms	3.97^**^ [1.55, 10.15]	<0.05	3.35^*^ [1.24, 9.07]	<0.05
Respiratory symptoms	1.29 [0.78, 2.12]	0.317	1.74^*^ [1.06, 2.84]	<0.05
Skin conditions	2.02 [0.70, 5.76]	0.191	3.69^*^ [1.06, 12.85]	<0.05
Others	53.81^***^ [7.05, 410.65]	<0.05	11.95^***^ [5.08, 28.15]	<0.05

Estimates were adjusted for age, gender, parental living status, person taking care, where living, type of family, number of family members living with a child, whether a child is going to school/College, marital status, and the wealth index.

AOR, Adjusted Odds Ratio. *p < 0.05, **p < 0.01, ***p < 0.001.

Multivariable logistic regression identified several factors associated with the presence of any acute morbidity at both baseline (Visit 1) and endline (Visit 8) (Table 7). CLHIV were significantly associated with higher morbidity at both baseline (AOR 1.99, 95% CI: 1.19–3.32, <0.05) and endline (AOR 2.76, 95% CI: 1.62–4.71, *p* < 0.001). Older age was protective, with lower odds among children aged 15–19 years and ≥20 years compared to 5–9 years at baseline (AOR 0.31 and 0.086) and endline (AOR 0.41 and 0.10). Non-attendance at school was associated with higher morbidity at baseline (AOR 2.16, <0.05) and endline (AOR 2.19, <0.05). Children from the richest households consistently demonstrated lower odds of morbidity throughout the study, with significant protective associations observed at the baseline (Visit 1) assessment (AOR 0.29, <0.05) and the final follow-up (Visit 8) assessment after 24 months (AOR 0.26, <0.05). Overall, HIV-positive status and lack of school attendance increased morbidity risk, while older age and higher wealth were protective.

**Table 7:** Association between selected participant characteristics and the presence of any morbidity at baseline and end-line visits in the prospective paediatric HIV cohort.

Variables	Baseline		End line	
	AOR	*p* value	AOR	*p* value
HIV status				
Negative (Ref.)				
Positive	1.99^**^ [1.19, 3.32]	<0.05	2.76^***^ [1.62, 4.71]	<0.05
Age				
5–9 (Ref.)				
10–14	0.53 [0.27, 1.06]	0.072	0.747 [0.37, 1.50]	0.414
15–19	0.31^**^ [0.13, 0.70]	<0.05	0.41^*^ [0.18, 0.95]	<0.05
20+	0.086^***^ [0.03, 0.29]	<0.05	0.10^***^ [0.03, 0.33]	<0.05
Sex of the Child				
Male (Ref.)				
Female	1.03 [0.63, 1.71]	0.899	1.23 [0.74, 2.06]	0.429
Parent living status				
Mother and father alive (Ref.)				
Mother deceased	4.29 [0.41, 44.93]	0.224	1.79 [0.19, 16.88]	0.612
Father deceased	0.99 [0.55, 1.79]	0.975	0.93 [0.50, 1.71]	0.811
Both mother and father deceased	8.90^*^ [1.50, 52.93]	<0.05	1.03 [0.19, 5.70]	0.970
Person taking Care				
Mother (Ref.)				
Father	0.38 [0.03, 4.80]	0.455	0.91 [0.08, 10.17]	0.941
Other persons	0.12^*^ [0.02, 0.72]	0.021	0.72 [0.13, 4.11]	0.714
Where living				
With mother at home (Ref.)				
Without mother at home	1.65 [0.55, 4.93]	0.367	1.06 [0.34, 3.30]	0.914
Other Places	2.11 [0.62, 7.11]	0.23	0.37 [0.11, 1.29]	0.118
Type of family				
Nuclear (Ref.)				
Joint	0.83 [0.39, 1.76]	0.621	0.49 [0.22, 1.06]	0.071
No. of family members living with the child				
Less than or equal to 4 (Ref.)				
Above 4	1.38 [0.78, 2.44]	0.267	1.19 [0.66, 2.15]	0.563
Whether the child is going to school/college				
Yes (Ref.)				
No	2.16^*^ [1.02, 4.59]	<0.05	2.19^*^ [1.01, 4.76]	<0.05
Marital status				
Married (Ref.)				
Unmarried	1.73 [0.50, 6.00]	0.385	1.21 [0.34, 4.38]	0.767
Wealth index				
Q1-Poorest (Ref.)				
Q2-Poorer	0.42^*^ [0.19, 0.97]	<0.05	0.39^*^ [0.17, 0.91]	<0.05
Q3-Middle	0.42^*^ [0.21, 0.82]	<0.05	0.38^**^ [0.19, 0.75]	<0.05
Q4-Richer	0.55 [0.25, 1.20]	0.135	0.67 [0.29, 1.51]	0.332
Q5-Richest	0.29^**^ [0.13, 0.65]	<0.05	0.26^**^ [0.11, 0.60]	<0.05

AOR, Adjusted Odds ratio. *p < 0.05, **p < 0.01, ***p < 0.001.

### Survival probability among 372 children

Survival analysis was conducted among 372 children followed over 24 months, during which a total of four deaths were recorded. The estimated one-year survival probability was 0.99 (95% CI: 0.98–1.00), which declined marginally to 0.98 (95% CI: 0.97–0.99) at the end of 2 years. Most deaths occurred early during follow-up, with two deaths by the second visit and one additional death each at the fifth and sixth visits. Kaplan–Meier survival curves demonstrated a high overall probability of survival throughout the study period, with a gradual decline over successive visits (Table 8). Stratified survival curves by HIV status showed consistently lower survival probabilities among HIV-positive children compared with HIV-negative children, although survival remained high in both groups across follow-up (Figure 2).

**Table 8:** Distribution of deaths and Kaplan–Meier survival probabilities by follow-up visit among children in the prospective paediatric HIV cohort.

Visit	Death	Alive	Survival probability	95% CI
1	0	372	1	–
2	2	370	0.9946	(0.9876–1.0000)
3	0	370	0.9946	(0.9876–1.0000)
4	0	370	0.9946	(0.9876–1.0000)
5	1	369	0.9919	(0.9830–1.0000)
6	1	368	0.9892	(0.9789–0.9996)
7	0	368	0.9892	(0.9789–0.9996)
8	0	368	0.9892	(0.9789–0.9996)
Total	4			

CI, Confidence Interval.

**Figure 2:**
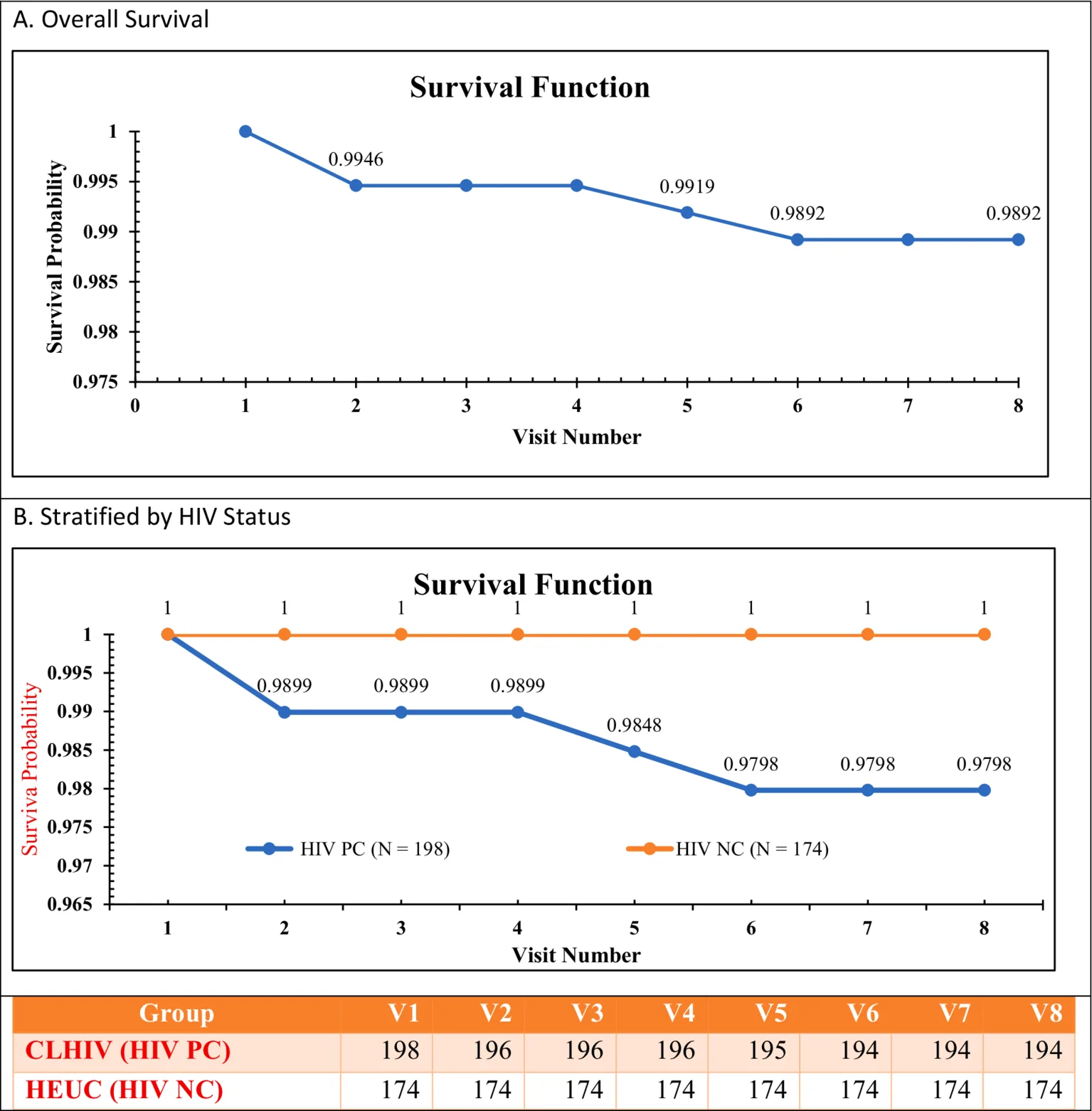
Kaplan–Meier survival curves showing the probability of survival among 372 children during follow-up in the prospective paediatric HIV cohort. **(A)** Overall survival. **(B)** Survival stratified by HIV status, showing children living with HIV (CLHIV; *n* = 198) and HIV-exposed uninfected children (HEUC; *n* = 174).

Among 348 children, 5 (1.44%) had TB; 343 (98.56%) had no history. All cases had one episode. Pulmonary DSTB was seen in 4 (80.0%) and extrapulmonary DSTB in 1 (20.0%). Confirmation was obtained in 2 (40.0%); 3 (60.0%) were diagnosed clinically and/or radiologically (Figure 3).

**Figure 3:**
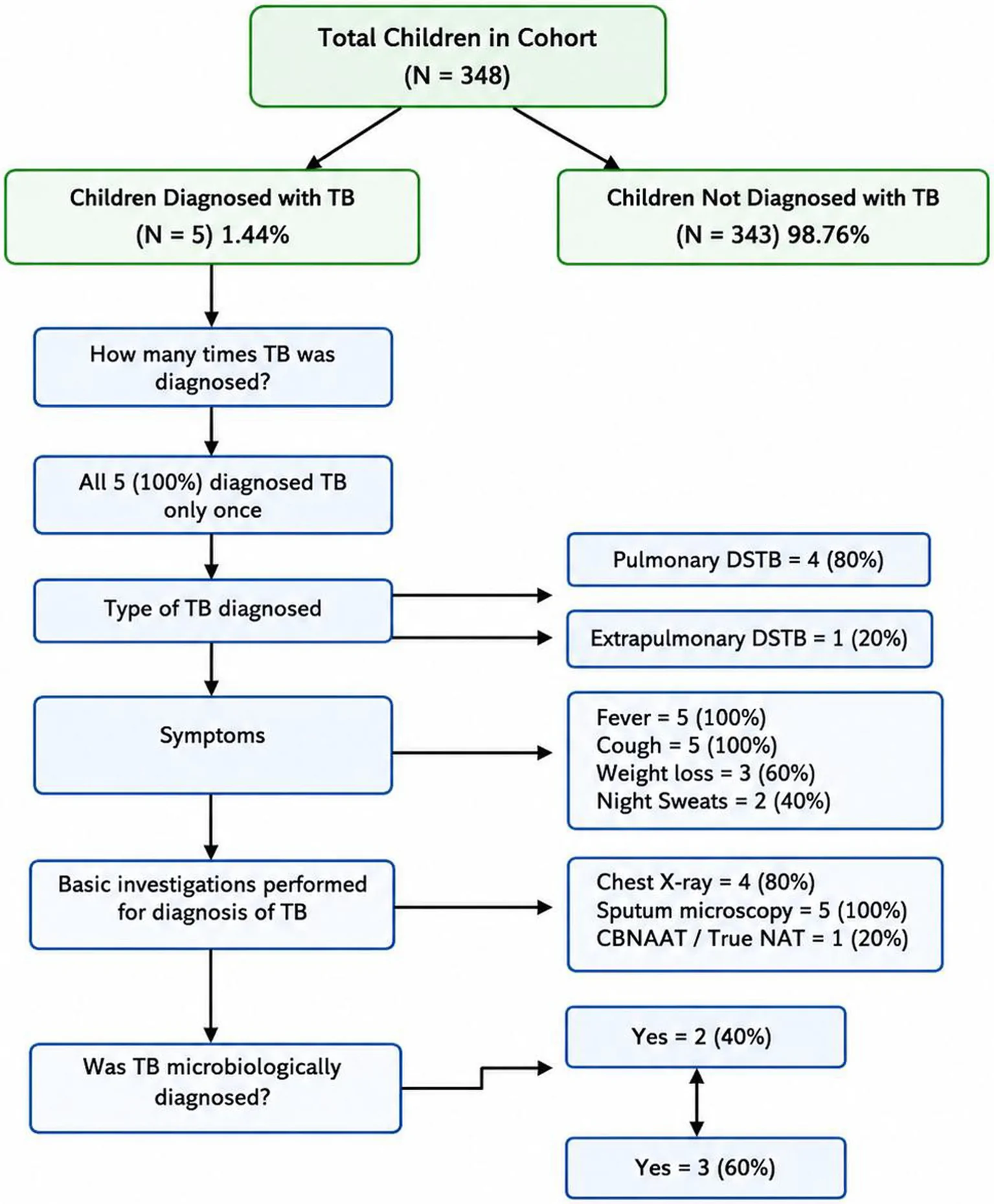
Tuberculosis-related characteristics and outcomes among children enrolled in the prospective paediatric HIV cohort.

## Discussion

Paediatric HIV infection remains a significant global health challenge, particularly in low- and middle-income countries (LMICs), despite major advances in prevention of mother-to-child transmission (PMTCT) and antiretroviral therapy (ART) (16).

The findings from this cohort study are consistent with previous ICMR task force research conducted in Belagavi district, which demonstrated a substantial, although declining, burden of paediatric HIV infection and associated morbidities. Collectively, these studies reinforce the importance of continuous surveillance and evidence-based interventions to reduce HIV transmission and improve health outcomes among children. CLHIV continue to experience a substantial burden of comorbidities related to chronic immune activation, delayed ART initiation, opportunistic infections, ART-associated toxicities, and adverse socioeconomic conditions. The findings of the present study are consistent with previous reports demonstrating that co-morbid conditions persist even in the era of widespread ART availability (16–18).

HIV-related immunosuppression increases susceptibility to both pulmonary and extrapulmonary TB, while TB infection may accelerate HIV disease progression and complicate treatment outcomes (17). Recurrent bacterial infections, including pneumonia and sepsis, have also been widely documented and are often associated with advanced disease, malnutrition, and incomplete immunization coverage (16, 19).

In the present cohort, respiratory infections were the most common morbidity, accounting for 37% of the reported morbidities, which is consistent with findings from a study conducted by Rabie and Goussard (20). The incidence of respiratory infections was also highest among HEUC participants in our cohort. The higher occurrence of respiratory infections may be related to impaired cell-mediated immunity and increased susceptibility to opportunistic infections among children living with HIV (21).

While our study found significantly higher odds of gastrointestinal symptoms among CLHIV compared with HEUC, the overall prevalence of diarrhoeal illness remained notable in both groups. This finding is consistent with a meta-analysis of studies published between 1993 and 2018, which reported a higher risk of diarrhoea among CLHIV than HEUC (22). Malaria was the second most common morbidity observed in our cohort. The high burden of both diarrhoeal illness and malaria may reflect the local epidemiological context, including the endemicity of vector-borne diseases and other infectious conditions that may disproportionately affect children living with HIV. Similarly, a study conducted in southern Morocco reported diarrhoea as the most common opportunistic infection among children living with HIV, followed by tuberculosis and respiratory infections (21).

Previous studies from Ethiopia, India, and Latin America have reported varying rates of tuberculosis among people living with HIV, ranging from approximately 11 to 35% (23–26). In Ethiopia, TB was reported in 29% of children living with HIV, while an Indian study reported TB as the most frequent infection, accounting for 35% of infections (23–25). Similarly, Dravid et al. (26) reported a TB incidence of 22% among HIV-infected individuals receiving long-term antiretroviral therapy at ART clinics in Pune, Western India. In contrast, the current investigation observed a considerably lower incidence of TB (1.5%). A 2024 study across eight Latin American countries also reported TB in 11% of children living with HIV who developed opportunistic infections (27). The variation in TB occurrence across studies may reflect differences in study populations, geographical settings, study designs, diagnostic approaches, duration of follow-up, and access to antiretroviral therapy and TB preventive interventions. In a study conducted in western China, the TB/HIV co-infection group male-to-female ratio was 2:1, higher than the TB-only group, 1.19:1. The highest proportion of TB/HIV co-infection was in the 5–9 years age group (43.6%), while the 10–14 years age group accounted for the highest proportion of TB-alone cases (57.5%) (28). The comparatively low TB incidence in the present cohort may reflect differences in regional TB burden, effectiveness of TB screening and preventive strategies, or variations in study design and diagnostic criteria. It also suggests that despite the global burden of TB among children living with HIV, local epidemiology can vary significantly.

Survival analysis was conducted among 372 children followed over 24 months, during which a total of four deaths were recorded. The estimated one-year survival probability was 0.99 (95% CI: 0.98–1.00), which declined marginally to 0.98 (95% CI: 0.97–0.99) by 2 years. A study conducted in Lesotho observed that the mortality in the HIV paediatric cohort during a 5.9-year follow-up was 6.1%. In their study, a maximum of 89% of deaths occurred in children under 5 years old (29). Younger children living with HIV may be at greater risk of morbidity and mortality because of their increased susceptibility to infections and immature immune responses (30). In a study conducted in Asia (31), a decade ago, the mortality in these children was 6.6%. Since the NACP’s implementation in our country, we have observed a significant reduction in parent-to-child transmission and mortality during adolescence.

The high burden of comorbidities among CLHIV highlights the need for a comprehensive, child-centred model of care that extends beyond viral suppression alone (17). Routine screening for infectious and non-infectious comorbidities, early neurodevelopmental assessment and mental health support should be integrated into paediatric HIV services (19, 31). Early diagnosis and prompt ART initiation remain essential to reducing long-term complications, but interventions addressing social factors associated with health are equally critical for improving overall outcomes (16, 17).

### Strengths

A key strength of this study is its prospective, community-based cohort design, which allowed longitudinal observation of morbidity patterns and survival over 2 years, providing important insights into the health trajectory of CLHIV and HEUC. Another important strength is the inclusion of a relatively older cohort than is traditionally studied in paediatric HIV research, providing an opportunity to describe morbidity patterns and longer-term health outcomes associated with HIV exposure and infection beyond early childhood.

### Limitations

Information on acute morbidity episodes was obtained from caregiver reporting during follow-up visits, which could have led to recall-related inaccuracies in reporting illnesses. Moreover, since the study was conducted in a particular geographic region, the findings may not be fully generalisable to other populations or settings. Some morbidity categories, particularly skin conditions and other conditions, had relatively few events, resulting in wider confidence intervals and reduced precision of the corresponding adjusted estimates; therefore, these findings should be interpreted with appropriate caution. Although the cohort was relatively large, the sample size may have limited the statistical power to detect differences for some secondary outcomes, particularly within smaller subgroups. Furthermore, multiple tests of association were performed; therefore, findings beyond the primary endpoint should be interpreted cautiously, particularly where estimates had wide confidence intervals.

## Data Availability

The raw data supporting the conclusions of this article will be made available by the authors, without undue reservation.
